# From cumulative cultural transmission to evidence-based medicine: evolution of medicinal plant knowledge in Southern Italy

**DOI:** 10.3389/fphar.2015.00207

**Published:** 2015-09-30

**Authors:** Marco Leonti, Peter O. Staub, Stefano Cabras, Maria Eugenia Castellanos, Laura Casu

**Affiliations:** ^1^Department of Biomedical Sciences, University of CagliariCagliari, Italy; ^2^Department of Mathematics and Informatics, University of CagliariCagliari, Italy; ^3^Department of Statistics, Carlos III University of MadridGetafe, Spain; ^4^Department of Informatics and Statistics, Rey Juan Carlos UniversityMóstoles, Spain; ^5^Department of Life and Environmental Sciences, University of CagliariCagliari, Italy

**Keywords:** traditional medical knowledge, cultural transmission and evolution, causal effect, evidence-based, herbal medicine, historical ethnopharmacology, *De Materia Medica*, globalization

## Abstract

In Mediterranean cultures written records of medicinal plant use have a long tradition. This written record contributed to building a consensus about what was perceived to be an efficacious pharmacopeia. Passed down through millennia, these scripts have transmitted knowledge about plant uses, with high fidelity, to scholars and laypersons alike. Herbal medicine's importance and the long-standing written record call for a better understanding of the mechanisms influencing the transmission of contemporary medicinal plant knowledge. Here we contextualize herbal medicine within evolutionary medicine and cultural evolution. Cumulative knowledge transmission is approached by estimating the causal effect of two seminal scripts about *materia medica* written by Dioscorides and Galen, two classical Greco-Roman physicians, on today's medicinal plant use in the Southern Italian regions of Campania, Sardinia, and Sicily. Plant-use combinations are treated as transmissible cultural traits (or “memes”), which in analogy to the biological evolution of genetic traits, are subjected to mutation and selection. Our results suggest that until today ancient scripts have exerted a strong influence on the use of herbal medicine. We conclude that the repeated empirical testing and scientific study of health care claims is guiding and shaping the selection of efficacious treatments and evidence-based herbal medicine.

## Introduction

In contrast to the relatively homogenous human genome, human culture is characterized by diversity (Pagel and Mace, 2004). The origin of our cultural diversity and the question as to which parameters influence its dynamics are issues central to population genetics, anthropology and evolutionary biology (Guglielmino et al., 1995; Henrich and Boyd, 1998; Pagel and Mace, 2004). Human cultures and their persistence are grounded in the ability of individuals to learn from and copy each other in a process essential for cultural evolution termed “cultural transmission” (Cavalli-Sforza et al., 1982; Lehmann et al., 2010; Pennisi, 2010). While social learning and copying of cultural traits adds to the evolutionary success of animal species, particularly humans (Laland and Janik, 2006; Rendell et al., 2010), human cultures differ from other primate cultures by evolving constantly and cumulatively, such that waves of innovations and trait modifications become continuously assimilated (Tennie et al., 2009). This essential difference has been explained by humans having a “theory of mind,” an ability to adopt another's point of view and understand the intentionality and purpose of action, and hence to innovate useful modifications to them, instead of the mindless copying that most non-human species engage in (Tomasello, 1999).

“Cultural traits” (Cavalli-Sforza and Feldman, 1981, p. 70) are units of transmittable knowledge hierarchically organized by scales of complexity and inclusiveness (O'Brien et al., 2010). Cultural change occurs when, in analogy to genetic evolution, traits are affected by mutation (incorrect knowledge transmission, loss of knowledge or traits, creation, and assimilation of new traits), recombination (mixture of traits), cultural drift (random processes) and guided by natural selection (Mesoudi et al., 2004; O'Brien et al., 2010; Cardoso and Atwell, 2011). Quantitative observations in cultural transmission are restricted to the study of cultural traits, which may also be described as units of cultural replicators propagated through imitation and termed “memes” (Dawkins, 1976, p. 189 ff.). With the concept of “memes” seemingly irrational behavior and religion can be equally well accounted for as technically complex recipes, whereas the success rate of memes or cultural traits is determined by their ability to spread between and lodge themselves in human minds and cultures (Dawkins, 1976; Strimling et al., 2009). Oblique knowledge transmission describes the passing down of cultural traits by members of one generation to extra-familial members of the next generation. A special case of oblique transmission that increases cultural homogeneity, results from teacher-pupil relationships (Cavalli-Sforza and Feldman, 1981, p. 54; Cavalli-Sforza et al., 1982). Also exclusive to humanity is the ability to transmit knowledge by means of language and symbology, via media such as scripts, art forms and telecommunication to subjects remote in space and time (Cavalli-Sforza and Feldman, 1981, pp. 3–4). The transmission of knowledge through print media results in a more precise and detailed passing down of information and hence dissemination of cultural traits (Diamond, 2005, p. 216). External storage of human knowledge, such as scripts, can act as interregional repositories and influence technological change, increase high-fidelity transmission, and preserve knowledge. Repositories increase the longevity as well as the diversity of cultural traits within a cultural group (Lewis and Laland, 2012; Mesoudi et al., 2013). The analysis of cultural transmission has been approached with the aid of proxies, such as archeological artifacts (e.g., O'Brien et al., 2010), or by means of animal or human behavioral experiments (Tennie et al., 2009; Rendell et al., 2010), phylogenetic inference (e.g., Barbrook et al., 1998) and using mathematical models (e.g., Strimling et al., 2009; Lehmann et al., 2010; Nunn et al., 2010; Lewis and Laland, 2012). Relative rarely knowledge transmission has been approached through statistical analyses of data obtained by means of real observations (but see: Hewlett and Cavalli-Sforza, 1986; Reyes-García et al., 2009; Leonti et al., 2010; Soldati et al., 2015).

Human knowledge about medicinal traditions and practices is well documented and can be traced back to the earliest writing, offering possibilities for diachronic studies (e.g., Heinrich et al., 2006; Pollio et al., 2008; Dal Cero et al., 2014). Historically, medicinal plants and their products have quantitatively dominated *materia medicae* and pharmacopeias. A pharmacopeia is a standard recipe book describing the preparation, formulation and application of medicines. The usefulness of a pharmacopeia is “determined by the periodical changes it has to undergo to keep pace with the latest progress in the sciences on which it is based” (Urdang, 1951, p. 577). Nonetheless, since medicinal plant knowledge and traditional medicine are at once adaptive yet deeply rooted in local traditions and history they show both conservative and progressive characters (Leslie, 1976, p. 1–17; Bye et al., 1995; Leonti, 2011). In modern societies and urban centers herbal medicine is frequently chosen as a treatment for mild or chronic ailments and as an adjuvant therapy. In rural and deprived areas, however, herbal medicine frequently constitutes the only affordable treatment option (Leonti and Casu, 2013). Potentially, any plant or natural product can be used as a medicine and answers to questions such as “what is an accepted medicinal plant?” and “how many different plants are globally being used as medicines?,” depend on the applied consensus or definition. The Kew Medicinal Plant Names Services currently catalogs around 13'500 medicinal plant species worldwide^1^.

In general, cultural interactions (including factors such as exchange of biodiversity, associated knowledge, epidemics and political hegemony) can affect the continuity of medicinal plant use and may lead to recombination of traits and innovation. “Disjunction” describes a changing ethnomedical context applied to original remedies, “discontinuity” the giving up of a plant use and “synchronism” the substitution of a native species by a hitherto not considered species, or by introduced plants with similar semantic backgrounds (Bye et al., 1995). Medicine is, however, culture bound and includes rational (empirical) as well as irrational (symbolic) aspects and behavior. The placebo effect, or meaning response, for example, is a physiologically poorly described phenomenon and conceptualizes how subjective perception, expectation and cultural meaning influences the effectiveness of medicinal treatments (Etkin, 1988; Moerman and Jonas, 2002; Rief et al., 2011). Today, complementary and traditional medicines hold a multi-billion dollar market-share. Also therefore, it is important to understand the cultural dynamics and factors that influence the transmission of efficacious vs. non-efficacious medical treatments (Tanaka et al., 2009).

We and others have argued that scripts reporting and approving therapeutical uses of plants and remedies in general, may act as blueprints. Scripts facilitate high fidelity knowledge transmission and thereby shape the cultural and inter-cultural use of plant-based medicines (Leonti et al., 2009, 2010; Brown et al., 2014). The European Pharmacopeia and the use of herbal medicine have been influenced considerably by the Greco-Roman medical texts, their medieval Arabic interpretations, as well as by the Renaissance commentaries (Urdang, 1951; Mann, 1984; Heinrich et al., 2004). Dioscorides' and Galen's works were among the first printed medicinal texts, and Pietro Andrea Matthioli's translation of Dioscorides' *De Materia Medica*, remained the fundamental pharmacological text in Italy until the eighteenth century (Cosmacini, 2009). This well documented historical development, together with the wealth of historical records on *materia medicae*, provide a framework conductive to the quantitative analysis of high fidelity knowledge transmission of medical plant use, and the process of trait evolution.

Here we use causal inference, which is a statistical perspective designed to analyze the existence of causal connections between two categories of variables. The units of analysis are citations of plant use in herbal books and independent field studies, quantitatively arranged into medicinal use-categories. These plant taxon-use-category pairs are treated as cultural traits, compared, and analyzed with Bayesian statistical inference, and in particular with the Bayesian Additive Regression Trees (BART) model. Our aim is to determine the causal effect of the therapeutical recommendation of Dioscorides (first century AD) and Galen (ca. 130–200 AD) on contemporary (1970–2013) medicinal plant use in the Southern Italian regions of Campania, Sardinia and Sicily. *De Materia Medica* (henceforth *DMM*) written by Dioscorides probably in the second half of the first century AD, and Galen's *De simplicium medicamentorum facultatibus libri XI* (henceforth *DSMF*), written during the second half of the second century AD, are among the most copied and influential texts on herbal medicine in history (Singer, 1927; Arber, 1953; Riddle, 1985). The regions of Campania, Sardinia, and Sicily have experienced similar cultural impacts to varying degrees. While parts of Campania and Sicily belonged to Magna Graecia (800 BC onwards), after the first Punic war (264–241 BC) all three regions were absorbed by the Roman Empire (Saitta, 1967; Palmer, 1977). Greek, however, remained lingua franca in Southern Italy until the sixth century AD what facilitated the transmission of classical knowledge to later ages. The estimation of causal effects of historic therapeutical plant recommendations over contemporary medicinal plant knowledge is a problem of causal inference. The “causal effect” is determined by asking “how would the contemporary plant uses change (i.e., increase or decrease of specific contemporary plant-use combinations) if the authors (Dioscorides and Galen) had given the opposite indication?”.

Addressing this research question is crucial for fostering the link between empirical “traditional” knowledge and biomedicine (see Etkin, 2001 for a comprehensive discussion): First, because it helps to understand the role and importance of texts in the transmission of medicinal plant knowledge and the development of pharmacopeias overall. Second, because it allows a critical discussion of the relevance of contemporary field surveys aiming at contributing to natural products research and conserving “traditional” knowledge in regions with a pronounced written tradition. We have approached this question before for the region of Campania with a limited set of data (Leonti et al., 2010), but now include a cross-cultural analysis considering two additional South Italian regions including all commonly used medicinal plant taxa described in Dioscorides' *DMM* and Galen's *DSMF*.

## Materials and methods

### Data sources and sampling

Matthioli's (1501–1578) translation of Dioscorides' *DMM* from 1568 (reedited as a facsimile in 1967–1970) and the Latin-Greek transcription of Galen's *De simplicium medicamentorum facultatibus libri XI* (*DSMF*) by Theodorico Gerardo Gaudano, published by Gulielmum Rouillium (Guillaume Rouillé) in 1561 were used for extracting the historical therapeutical indications of medicinal plant taxa. These historical indications are treated as the influencing information. With respect to *DMM* and our previous analysis (Leonti et al., 2010) where we also included the recommendations made by Matthioli himself, here we only consider the text attributed to Dioscorides (*ex* Matthioli, 1568).

**Figure 1:**
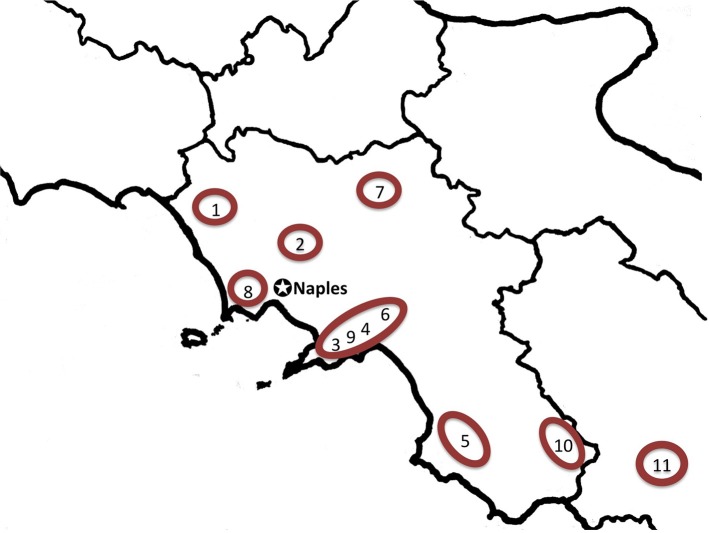
**Map of Campania and adjacent regions indicating considered field studies addressing popular medicinal plant use**. 1, Roccamonfina, Caserta (Antonone et al., 1988); 2, Caserta, Caserta (De Feo et al., 1991); 3, Peninsula Sorrentina, Napoli/Salerno (De Feo et al., 1992); 4, Coast of Amalfi, Salerno (De Feo and Senatore, 1993); 5, Monte Vesole and Ascea, Salerno (Scherrer et al., 2005); 6, Montecorvino Rovella, Salerno (De Natale and Pollio, 2007); 7, Sannio area, Benevento (Guarino et al., 2008); 8, Phlegraean Fields Regional Park (Motti et al., 2009); 9, Amalfi Coast (Savo et al., 2011); 10, National Park of Cilento and Vallo di Diano (Di Novella et al., 2013); 11, Rotonda, Pollino National Park (Di Sanzo et al., 2013).

**Figure 2:**
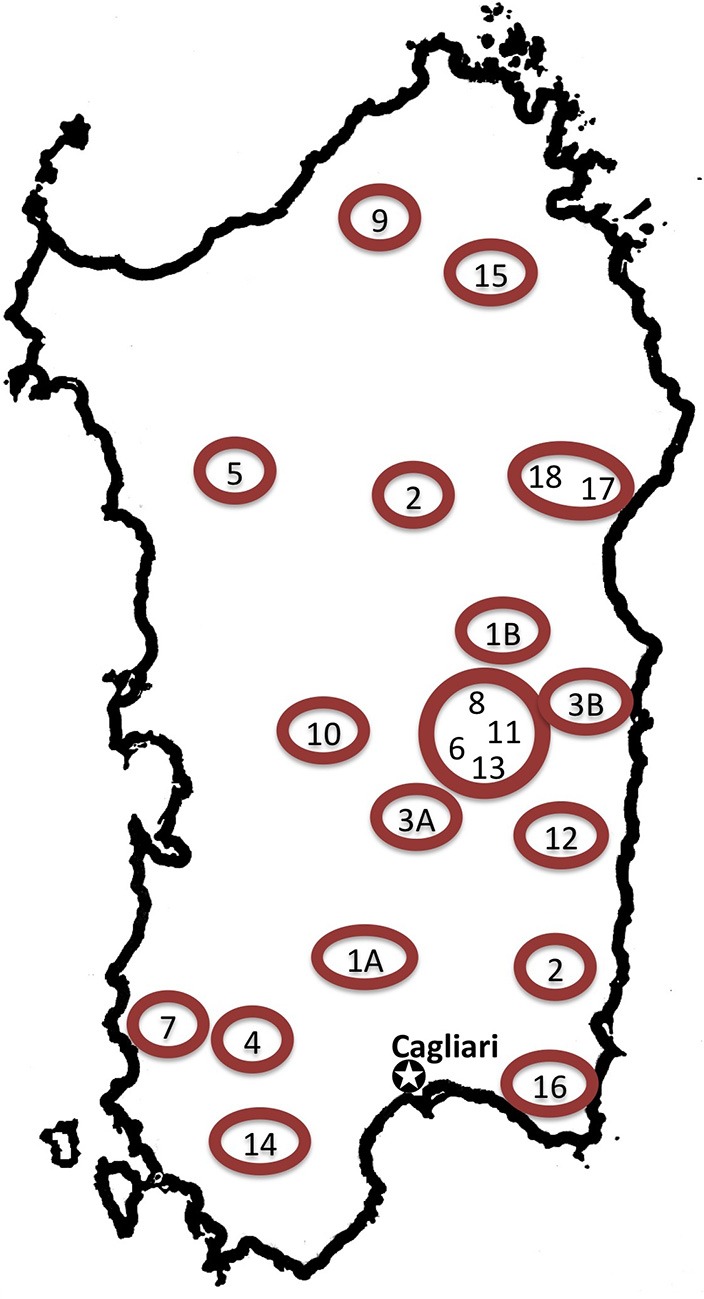
**Map of Sardinia indicating considered field studies addressing popular medicinal plant use**. 1A, Campidano; Cagliari (Bruni et al., 1997); 1B, Urzulei, Ogliastra (Bruni et al., 1997); 2, Sarrabus, Cagliari (Palmese et al., 2001); 3A, Escolca, Cagliari (Loi et al., 2005); 3B, Lotzorai, Ogliastra (Loi et al., 2005); 4, Marganai, Carbonia-Iglesias (Ballero and Fresu, 1991); 5, Monteleone, Sassari (Ballero and Poli, 1998); 6, Seui, Ogliastra (Ballero and Fresu, 1993); 7, Fluminimaggiore, Carbonia-Iglesias (Ballero et al., 2001); 8, Villagrande Strisaili, Ogliastra (Loi et al., 2004); 9, Tempio Pausania, Olbia-Tempio (Ballero et al., 1997); 10, Laconi; Oristano (Ballero et al., 1997); 11, Arzana, Nuoro (Ballero et al., 1994); 12, Perdasdefogu, Ogliastra (Ballero et al., 1997); 13, Ussassai; Ogliastra (Ballero et al., 1998); 14, Carbonia-Iglesias (Atzei et al., 1994); 15, Gallura; Olbia-Tempio (Atzei et al., 1991); 16, Villasimius, Cagliari (Ballero, 1982); 17, Dorgali (Camarda, 1990); 18, Monte Ortobene, Nuoro (Signorini et al., 2009).

**Figure 3:**
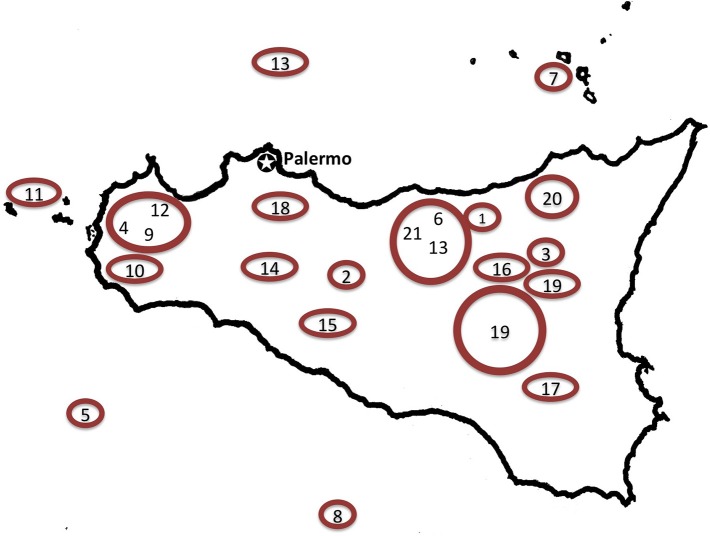
**Map of Sicily indicating considered field studies addressing popular medicinal plant use**. 1, Mistretta; Messina (Lentini and Raimondo, 1990); 2, Mussomeli; Caltanissetta (Amico and Sorge, 1997); 3, Cesarò; Messina (Barbagallo et al., 1979); 4, Erice; Trapani (Lentini and Aleo, 1991); 5, Pantelleria; Trapani (Galt and Galt, 1978); 6, Madonie, Palermo (Raimondo and Lentini, 1990); 7, Eolie, Messina (Lentini et al., 1995); 8, Pelagie, Agrigento (Lentini et al., 1996); 9, Trapani (Lentini, 1987); 10, Mazara del Vallo; Trapani (Lentini et al., 1987–1988); 11, Egadi; Trapani (Lentini et al., 1997); 12, Riserva Naturale Dello Zingaro; Trapani (Lentini and Mazzola, 1998); 13, Ustica; Palermo (Lentini et al., 1994); 14, Bivona, Agrigento (Catanzaro, 1970); 15, Sant'Angelo Muxaro, Agrigento (Lentini, 1996); 16, Bronte, Catania (Arcidiacono et al., 1999); 17, Monterosso Almo, Ragusa (Napoli and Giglio, 2002); 18, Mezzojuso, Palermo (Ilardi and Raimondo, 1992); 19, Sicilia centro-orientale (Barbagallo et al., 2004); 20, Alcara Li Fusi e Militello Rosmarino, Messina (Arcidiacono et al., 2007); 21, Madonie Regional Park (Leto et al., 2013).

Data on contemporary medicinal plant use were compiled from 52 ethnobotanical studies on local medicinal plant use in the Italian regions of Campania (*n*_*c*_ = 11 study sites, including 1 study from the adjacent Basilicata), Sardinia (*n*_*s*_ = 20 study sites) and Sicily (*n*_*si*_ = 21 study sites) published between 1970 and 2013 (Figures 1–3; Supplementary Material, Supplementary Tables S1–S3). The taxa concertedly mentioned in Dioscorides' *DMM* (*ex* Matthioli, 1568), Galen's *DSMF* (1561), and in the contemporary studies conducted in Campania, Sardinia and Sicily, are included in this analysis. Closely related plant species used interchangeably and forming use-complexes generally perceived as ethnotaxa, are treated as one taxon (e.g., *Anemone* spp. includes *A. coronaria* L., *A. hortensis* L., and *A. nemorosa* L.). Species synonymies were resolved following theplantlist.org (The Plant List 1.1). For a complete list of species considered see Supplementary Material, Supplementary Table S4.

### Use-categories and cultural traits

The therapeutical indications of medicinal plants reported in Dioscorides and Galen, as well as those reported in the contemporary studies, were consistently allocated into 11 use-categories corresponding to organ or symptom-defined illness groups. Remedies and treatments of the eye, ear, and nose were classified as separate use-categories following Matthioli (1568) and Preuss (1971, pp. 300–341).

The eleven use-categories are: GAS, gastrointestinal disorders (including liver and spleen); URO, urological problems; RES, respiratory complaints (including angina, sore throat, pleurisy); DER, dermatologic problems (including oral cavity, varicose veins and hemorrhoids); SKM, skeleto-muscular disorders (including hematoma and gout); NER, central and peripheral nervous system (including headache, toothache, analgesic uses, epilepsy, insomnia); GYN, gynecology (application in women's medicine); FEV, fever, malaria; EYE, problems of the eye; EAR, problems of the ear; NOS, problems of the nose not related to respiratory diseases (epistaxis, polyps).

Each plant taxon-use-category pair is treated as a separate cultural trait (cf. Leonti et al., 2010) or “meme” based on which the causal influence of Dioscorides' *DMM* and Galen's *DSMF* on local South Italian contemporary medicinal plant use is determined. For each of the three regions, contemporary plant use is scored with the number of studies where a plant taxon-use-category pair was cited. For the region of Campania the scores could thus take the values *c*_*ij*_ = [0, 11], for Sardinia *s*_*ij*_ = [0, 20] and for Sicily *si*_*ij*_ = [0, 21] (Supplementary Material, Supplementary Tables S1–S3). Historical plant use is scored in a binary fashion where each plant taxon-use-category pair was either recommended or not mentioned by Dioscorides, *d*_*ij*_ = [0, 1] and by Galen *g*_*ij*_ = [0, 1].

### General statistical procedure

In general, and when not explicitly specified, for studying the association between two categorical variables we make use of the Chi-square test. This is a statistical test useful for assessing the significance of the association between two categorical variables, for example the recommendations of Dioscorides and Galen. The rest of the analyses that involve more variables and complicated relation structures have been performed with techniques explained below. We furthermore cross-check plant-use traits not present in Galen and Dioscorides with the content of a popular book on herbal medicine issued in 1980 by Reader's Digest called “Segreti e virtú delle piante medicinali” (Secrets of the properties of medicinal plants) (Reader's Digest, 1980).

### Probit regression

The probit regression is a regression model where the response variable is handled as a proportion. The proportion is transformed into a variable that varies all over the real line. Subsequently, this variable is used as the response variable in the usual linear regression model. The transformation of the proportion is achieved by inverting the cumulative distribution function of the standard Gaussian law. This function is called “probit function” wherefrom the name “probit regression” derives.

With the probit regression (e.g., Dobson and Barnett, 2008; Hastie et al., 2009) we estimate the overall similarities in citations of plant-use combinations (traits) with respect to the regions, and the joint citations of Dioscorides and Galen and the eventual interactions between such joint citations and regions. The latter is necessary in order to answer the questions of whether Dioscorides' and Galen's joint recommendations increased the overall trait similarity among regions. Citation proportions (i.e., trait proportions) are regressed against the five effects: 1. Geography (region), 2. Plant taxon, 3. Use-category, 4. Joint recommendations by Dioscorides and Galen and 5. Interaction between joint recommendations and geography. The fifth effect “Interaction between joint recommendations and geography” is the one we are focusing on.

### Causal inference

We use a statistical analysis, suitable for non-experimental settings in order to measure the evidence for a causal effect of Dioscorides and Galen upon contemporary indications on medicinal plant use. This analysis is based on data for all plants, use categories and regions, and the response variable *Y*. *Y* is the number of contemporary studies reporting a specific plant-use-category pair for a certain region. The minimum value of *Y* is 0, while its theoretical maximum value is 21 (Sicily). Variable *Y* represents the possible outcome, in terms of frequency of mentioned uses, which may has been caused by Dioscorides and Galen. The degree to which the outcome *Y* has been influenced by the two classic authors needs to be estimated with the BART model. The BART model takes into account that different other effects may have affected *Y*, and thus, possibly confound the relation between the two authors and the variable *Y*. These variables are referred to as “confounding variables” and denoted by *X*. We assume that the specific plant taxon, the specific therapeutical indication as well as geographical particularities can themselves be the cause of, or shape the outcome, independently of Dioscorides' or Galen's recommendations. Therefore, *X* includes the confounding variables (i) plant taxon, (ii) therapeutical use, and (iii) geography into the causal relation and the estimation of the causal effect of Dioscorides and Galen on the contemporary observations:

- *Plant taxon*: The plant taxon determines the content of secondary metabolites and associated pharmacological properties. Plant identity also influences organoleptic properties of herbal drugs, which can have an impact on the assigned therapeutical indication and the mode of application.
- *Therapeutical use*: Each health problem has its own probability to be cured with medicinal plants.
- *Geography*: The local cultural history, regional epidemiology and the regional abundance of a taxon (biogeography) influences the probability that a plant taxon is used for medicine.

**Figure 4:**
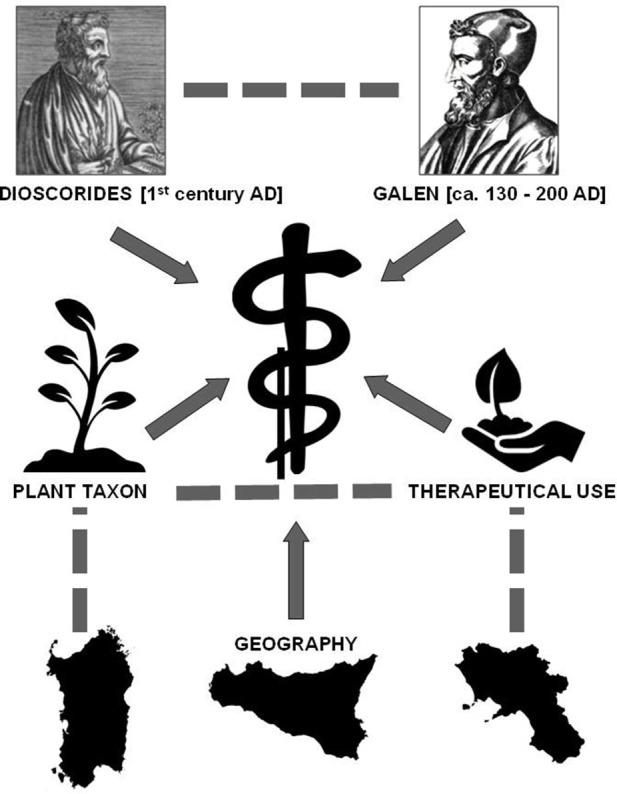
**Causal model assuming that Dioscorides and Galen influenced contemporary medicinal plant use considering the confounding variables “plant taxon,” “geography,” “therapeutical use” and their interactions (segments)**. Arrows indicate the direction of the influence, which may exert a causal effect.

We assume that Dioscorides as well as Galen influenced contemporary medicinal plant knowledge and that the influence of Galen on contemporary traits may itself be conditioned by the influence exerted by Dioscorides' work. The arrows in the causal model (Figure 4) indicate the direction of the influence, which may contain a causal effect.

Recommendations by Dioscorides and Galen are denoted by *Z*, where *Z* = 1 means that one or both authors made a recommendation for a certain combination of a plant-use-category. Both *X* and *Z* may affect *Y* and therefore, we need to separate the effect of *Z* from the confounding variables *X*. That is, we want to estimate the causal effect of *Z* on *Y*. The first principle of causal statistical inference is: “Correlation is not causation.” This means that whatever correlation we observe between *Z* and *Y*, it does not necessarily describe a causal effect, unless we assume that there are no other influential variables that determined the outcome *Y*. In fact, due to the existence of confounding variables we cannot measure the causal effects directly (see Figure 4 for the causal model).

The parameter of interest is α, i.e., the difference between the number of actually observed values of *Y* (contemporary plant-use combinations) and the potential values we would have observed if the author had given the opposite recommendation of *Z*. Since the outcome *Z* = 1 or *Z* = 0 was observed, the not observed outcome (*Z* = 0 or *Z* = 1) is referred to as the counterfactual outcome. This counterfactual outcome is what is estimated with the BART model (Chipman et al., 2010). In practice we estimate the causal effect based on the number of records per plant use combination in the contemporary literature by estimating the outcome of that number in the counterfactual sense and the theoretical situation where Dioscorides and Galen would not have recommend that use, although they did (Leonti et al., 2010). We do not consider the opposite situation, i.e., the counterfactual outcome of uses not recommended by Dioscorides of Galen because not mentioning a certain use is different from an explicit recommendation that a certain taxon should not be used for a certain application. In fact, cases where authors (i.e., physicians) did explicitly reject medicinal plant uses are rather rare. According to Matthioli (1568) Galen writes in the second chapter of the “Facoltà dei cibi” that *Siliquae* (the fruits of *Ceratonia siliqua*) are difficult to digest, and that it would have been better to leave them in the Orient, instead of bringing them into “our countries” (Matthioli, 1568, Book 1, Chap. 131, p. 258).

Moreover, we are not able to assess the causal effect of Dioscorides on Galen because we lack information on the confounding variables at that time. Finally, we conceive the causal effect α as a random variable, while the BART model provides the estimation of the probability distribution of α conditionally on the observed data. This estimation is known as the posterior distribution of the causal effect. It allows us to calculate the most probable causal effect, its mean value and also its existence by estimating the probability that α differs from 0. Further details on the BART model and its use in estimating causal effects can be found in Hill (2011).

## Results

### General data matrix

A comparison and consensus analysis between Dioscorides' *DMM* (*ex* Matthioli, 1568) and Galen's *DSMF* (1561), and the plant uses reported for Campania, Sardinia and Sicily, resulted in a set of 87 commonly mentioned medicinal taxa (Table 1). Dioscorides and Galen highly agree (*p* < 0.0001) recommending the same therapeutical uses for the large part of the plant taxa although Dioscorides makes reference to more uses. Especially with respect to the smaller use-categories such as “eyes,” “ears,” and “nose” but also regarding women's medicine and skeleto-muscular disorders Galen mentions considerably fewer plants compared to Dioscorides. For the 87 taxa under analysis we collected 462 use-citations from *DMM* and 236 use-citations from *DSMF*. From a total of 957 [11 × 87] theoretically possible plant-use combinations 470 are mentioned neither by Dioscorides nor by Galen, 211 are recommended by both authors, 251 exclusively by Dioscorides and 25 by Galen alone.

**Table 1:** **Plant taxa considered in this analysis derived from a consensus analysis between medicinal plants used in Campania, Sardinia, and Sicily, as well as those described in Dioscorides' ***DMM*** and Galen's ***DSMF*****.

**Taxon**	**Dioscorides' *DMM* (*ex* Matthioli, 1568), Book–Chap**.	**Galen's *DSMF* (1561)**	**GAS**	**DER**	**NER**	**SKM**	**GYN**	**RES**	**FEV**	**URO**	**EAR**	**EYE**	**NOS**
*Adiantum capillus-veneris* L.	Adianto (IV–138)	Adiantum (p. 366)	4	10	4	1	18	13	0	3	0	1	0
*Allium cepa* L.	Cipolla capitata (II–140)	Crommyon–Caepa (p. 464–465)	7	13	2	2	1	12	0	12	2	0	1
*Anagallis arvensis* L. s.l.	Anagallide (II–169)	Anagallis (p. 377)	0	3	1	0	0	5	0	0	0	1	0
*Anemone* spp.	Anthillide (III–147)	Anemone (p. 378–379)	1	2	3	1	1	0	0	1	0	0	0
*Apium* spp.	Apio (III–69)	Selinon–Apium (p. 519)	7	2	0	4	0	5	1	11	0	0	0
*Artemisia* spp.	Abrotano, Assenzo, Assenzo marino, Artemisia, Artemisia delle frondi sottili (I–46; III–24–26, 121, 122; V–37)	Abrotonon, Artemisia, Absinthium (p. 353-360, p. 385, p. 388–389)	23	10	4	4	2	8	5	2	0	3	0
*Arum* spp.	Aro (II–156, 157)	Arum (p. 384–385)	0	8	0	5	0	1	0	1	0	0	0
*Arundo* spp.	Canna (I–95)	Calamus phragmites–Canna (p. 432)	1	16	1	1	2	1	1	5	1	0	0
*Asparagus* spp.	Asparago (II–114)	Asparagus (p. 385–386)	5	0	2	3	0	2	0	19	0	0	0
*Asphodelus* spp.	Asphodelo–Hastula regio (II–159)	Asphodelus (p. 387)	0	25	0	3	0	4	1	2	0	0	0
*Avena* spp.	Vena, Bromo (II–85; IV–142)	Aegilops–Avena (p. 366)	4	4	1	1	0	2	0	4	0	0	0
*Brassica* spp.	Brassica, Napi, Rapa (II–102, 103, 111)	Crambe–Brassica (p. 459–460, p. 166)	7	8	1	5	2	7	1	1	0	0	0
*Calamintha nepeta* (L.) Savi s.l.	Calamintha (III–38)	Calamintha–Nepitha (p. 429–431, p. 334)	10	8	5	4	1	5	0	1	0	0	0
*Centaurium erythraea* Rafn. s.l.	Centaurea minore (III–7)	Centaurium minus (p. 422–443)	7	5	1	0	1	0	12	0	0	0	0
*Ceratonia siliqua* L.	Silique (I–131)	Ceratonia (p. 444)	9	1	0	0	0	12	0	1	0	0	0
*Ceterach officinarum* Willd. s.l.	Aspleno (III–145)	Asplenum (p. 386)	4	2	1	0	1	5	1	15	0	0	0
*Cichorium intybus* L. s.l.	Cichoria salvatica (II–121)	Seris–Cichorium (p. 519 &^*^)	26	2	1	3	0	0	3	9	0	0	0
*Convolvulus arvensis* L.	Helsine (IV–49)	Elxine (p. 413)	7	4	0	3	0	0	0	0	0	0	0
*Crataegus* spp.	Oxiacantha (I–103)	Oxyacanthos (p. 497)	7	3	13	2	2	2	5	4	0	0	0
*Cyclamen* spp.	Ciclamino (II–153)	Cyclaminos–Rapu (p. 465–467)	3	4	0	1	2	0	0	0	0	0	0
*Cydonia oblonga* Mill.	Cotogno (I–132)	Cydonia (p. 168)	7	5	2	0	0	3	1	0	0	0	0
*Cynara* spp.	Cardo (III–14)	Scolymus (p. 524)	14	1	0	0	0	1	0	2	0	0	0
*Cynodon dactylon* (L.) Pers.	Gramigna (IV–3)	Agrostis–Gramen (p. 362)	18	3	1	2	1	8	2	32	0	0	0
*Daucus carota* L. s.l.	Pastinaca salvatica (III–54)	Daucus–Staphylinus (p. 402–403)	8	6	2	0	3	5	0	7	0	1	0
*Ecballium elaterium* (L.) A. Rich.	Cocomero salvatico (IV–156)	Sicyos agrios–Cucumis agrestrs (p. 522)	8	3	3	2	1	0	2	0	0	0	0
*Equisetum* spp.	Coda di cavallo (IV–48, 49)	Hippuris–Cauda equina (p. 424)	4	11	0	3	0	3	0	17	0	0	3
*Ficus carica* L.	Fichi (I–146)	Syca–Ficus (p. 529–530)	7	22	1	1	1	18	0	1	0	0	0
*Foeniculum vulgare* Mill.	Finocchio (III–76)	Marathrum–Foeniculum (p. 479–480)	27	3	1	1	7	8	0	8	0	2	0
*Fumaria* spp.	Fumaria (IV–112)	Capnios–Fumus (p. 433)	9	7	2	0	1	2	0	3	0	0	0
*Hedera helix* L. s.l.	Hedera (II–170)	Cissos–Hedera (p. 449–450)	2	18	6	7	2	10	0	0	0	0	0
*Helichrysum italicum* (Roth) G. Don s.l.	Helichriso (IV–59)	Amarantum (p. 373)	2	4	2	3	0	7	0	0	0	0	0
*Helleborus* spp.	Elleboro nero (IV–153)	Elleborus–Veratrum (p. 412)	0	2	3	1	2	0	0	0	0	0	0
*Hordeum vulgare* L.	Orzo (II–78)	Crithe–Hordeum (p. 461)	7	3	0	0	0	9	0	3	0	1	0
*Hypericum* spp.	Asciro, Androsemo (III–166, 167)	Hypericum (p. 542)	5	28	4	8	1	5	0	3	0	0	0
*Juglans regia* L.	Noci (I–142)	Carya–Nuces (p. 436–437)	7	7	2	1	0	3	0	1	0	0	0
*Lactuca* spp.	Lattuca (II–125)	Thridax–Lactuca (p. 422)	7	11	10	2	2	1	0	2	0	1	0
*Laurus nobilis* L.	Lauro (I–35, 87)	Daphne arbor–Laurus (p. 403)	44	6	6	13	4	17	3	3	1	0	0
*Lavatera* spp.	Althea (III–158–160)	Ebiscus–Althea (p. 406–407)	8	9	0	1	0	9	0	1	0	2	0
*Linum usitatissimum* L.	Lino (II–94)	Linospermom–Lini semen (p. 475)	10	13	3	2	1	15	0	0	1	1	0
*Lonicera implexa* Aiton	Periclimeno (IV–15)	Periclymenos–Volucrum maius (p. 503)	3	2	1	3	1	2	0	2	0	0	0
*Malva* spp.	Malva (II–109)	Malache–Malva (p. 478–480)	40	36	8	1	5	24	2	14	0	5	0
*Marrubium vulgare* L.	Marrobio (III–113)	Prasium–Marrubium (p. 510–511)	12	3	4	7	4	13	7	0	0	0	0
*Matricaria chamomilla* L. & *Tanacetum* spp.	Anthemide, Camamilla, Parthenio (III–148, 149)	Anthemis aut Chamamelum (p. 380)	19	10	16	9	7	5	1	1	1	8	0
*Mentha pulegium* L.	Pulegio (III–31)	Glichon–Pulegium (p. 399, p. 334)	9	4	3	2	1	5	0	1	0	1	0
*Mentha* spp.	Menta, Sisembro (II–117; III–36)	Hediosmos–Menta (p. 418)	25	11	10	5	3	9	2	0	0	0	0
*Morus* spp.	Moro (I–144)	Morea–Morus (p. 488)	4	1	0	0	0	4	1	2	0	0	0
*Muscari racemosum* Mill. & *Leopoldia comosa* (L.) Parl.	Bulbo che si mangia, Bulbo che fa vomitare (II–160, 161)	Bulbos emeticos–Bulbus vomitorius (p. 394)	1	2	1	0	0	0	0	5	0	0	0
*Myrtus communis* L.	Mirto (I–129)	Myrrhine–Myrtus (p. 490–491)	12	14	3	2	2	9	0	4	0	1	0
*Nasturtium officinale* R. Br.	Sisembro acquatico (II–117)	Cardamum–Nasturtium (p. 435)	11	3	2	0	3	6	1	7	0	0	0
*Ocimum basilicum* L.	Basilico (II–130)	Ocimon (p. 550)	11	4	5	0	1	6	0	1	1	0	0
*Olea europaea* L.	Olivo salvatico (I–28, 117-121)	Elaea–Olea, Elaeon–Oleum (p. 407–411)	13	17	1	6	1	3	7	2	5	0	0
*Origanum* spp.	Origano, Maiorana, Sansucho (I–44; III–29, 42)	Origanus, Amaracon–Maiorana, Sampsycon–Maiurana (p. 498, p. 373, p. 518)	10	3	7	5	2	13	0	1	0	0	0
*Papaver rhoeas* L.	Papavero salvatico (IV–66)	Mecon–Papaver (p. 483–484)	2	2	30	1	0	12	1	0	0	0	0
*Papaver somniferum* L.	Papavero domestico (IV–67)	Mecon–Papaver (p. 483–485)	1	0	5	0	0	1	0	0	0	0	0
*Parietaria* spp.	Helsine (IV–88)	Elxine (p. 412–413)	27	26	8	10	1	9	3	31	1	1	0
*Petroselinum crispum* (Mill.) Fuss	Petroselino (III–72)	Petroselinum (p. 504)	14	5	5	1	8	1	0	10	2	1	1
*Pinus* spp.	Pino (I–71)	Pitys–Pinus (p. 507)	0	5	1	0	0	3	0	2	0	0	0
*Pistacia lensticus* L.	Lentisco (I–36, 37, 72)	Schinos–Lentiscus (p. 532)	6	14	4	4	0	7	2	0	0	0	0
*Pistacia terebinthus* L.	Terebintho (I–36, 73)	Terminthos–Terebinthus (p. 534)	2	4	2	2	0	4	0	1	0	0	0
*Plantago* spp.	Piantagine, Coronopo (II–115, 119)	Arnoglossum–Plantago (p. 383–384)	10	20	0	5	1	5	0	8	0	3	0
*Polygonum aviculare* L. s.l.	Poligono maschio (IV–3)	Polygonon–Seminalis (p. 508–509)	6	5	0	1	0	1	1	9	0	0	0
*Prunus* spp.	Ciregie (I–130)	Cerasus (p. 443–444)	6	0	0	3	0	5	0	7	0	0	0
*Prunus dulcis* (Mill.) D.A. Webb	Mandorle (I–31, 140)	Amygdala (p. 375–376)	8	3	0	0	0	5	0	0	0	0	0
*Prunus persica* (L.) Batsch	Pesco (I–132)	Melea persice–Malus persica (p. 486)	4	1	1	0	0	0	0	1	0	0	0
*Punica granatum* L.	Melagrano (I–128)	Rhoea–Malum granatum (p. 516, p. 169, p. 318)	11	1	1	1	2	2	1	2	0	0	0
*Ranunculus* spp. & *Ficaria verna* Huds.	Ranunculo, Batrachio, Chelidonia minore (II–166, 172)	Batrachium (p. 391)	0	6	1	5	0	1	0	0	0	0	0
*Ricinus communis* L.	Ricino (IV–165)	Cici–Ricinus (p. 446)	7	2	1	1	1	0	0	0	0	0	0
*Rosa* spp.	Rosa (I–39, 104, 111, 112)	Rhodos–Rosa, De rosaceo (p. 515–516, p. 134 ff.)	8	5	0	0	1	4	0	3	0	5	0
*Rosmarinus officinalis* L.	Rosmarino coronario (III–83)	Libanotides (p. 474)	22	6	9	8	2	13	0	3	0	0	0
*Rubus* spp.	Rovo (IV–39)	Batos–Rubus (p. 391–392)	19	28	1	0	2	6	0	5	0	1	0
*Rumex* spp.	Lapatio, Rombice (II–106)	Lapathum (p. 470)	6	13	2	1	0	1	2	5	0	0	0
*Ruscus* spp.	Rusco, Lauro Alessandrino (IV–148, 149)	Daphne herba (p. 403)	4	7	2	4	0	0	0	13	0	0	0
*Ruta* spp.	Ruta (III–47)	Perganon–Ruta (p. 505)	28	7	7	17	8	2	1	1	2	6	0
*Sambucus nigra* L.	Sambuco (IV–175)	Acte–Sambucus–Ebulus (p. 370)	13	15	8	14	0	13	2	5	2	9	0
*Senecio* spp.	Senecio (IV–99)	Herigeon (p. 420)	4	1	0	1	6	1	0	2	0	0	0
*Solanum nigrum* L.	Solatro hortolano (IV–73)	Trychnon (p. 539–541)	0	12	8	6	0	2	0	1	0	0	0
*Sonchus* spp.	Soncho (II–120)	Sonchus (p. 527)	8	7	2	2	0	1	0	4	0	0	0
*Tamus communis* L. (now: *Dioscorea communis* (L.) Caddick & Wilkin.)	Vite nera (IV–184)	Ampelos melana–Vitis nigra (p. 375)	0	1	1	10	0	0	0	1	0	0	0
*Thymus* spp.& *Thymbra capitata* (L.) Cav.	Tragorigano, Thimo, Serpillo (III –30, 39, 41)	Thymum (p. 423)	12	7	4	3	1	16	1	2	0	1	0
*Trigonella foenum-graecum* L.	Fiengreco (II–93)	Telis–Foenum graecum (p. 536)	1	1	1	0	1	2	0	1	0	0	0
*Triticum* spp.	Grano (II–77, 81)	Pyros–Triticum (p. 513)	4	10	4	3	1	3	0	0	0	0	0
*Tussilago farfara* L.	Tossilagine (III–120)	Bechium–Tussilago (p. 393)	0	8	1	1	0	9	1	0	0	0	0
*Ulmus* spp.	Olmo (I–93)	Ptelea–Ulmus (p. 511–512)	0	8	0	4	0	1	1	0	0	0	0
*Umbilicus* spp.	Ombelico Venere (IV–94)	Cotyledon–Umbilicus veneris (p. 458)	2	23	2	0	0	1	0	2	0	0	0
*Urtica* spp.	Ortica (IV–96)	Acalephe–Urtica (p. 368)	19	36	5	17	3	6	1	12	1	0	3
*Verbascum* spp.	Verbasco (IV–106)	Phlomos–Verbascum (p. 543–544)	3	16	2	0	0	8	0	1	0	0	0
*Verbena officinalis* L.	Verbenaca (IV–62)	Peristereon–Verbena (p. 503)	6	6	6	7	1	3	8	0	0	0	0
Total			759	713	273	262	128	455	84	346	20	55	8

*Red, Dioscorides and Galen recommend the use; Yellow, Only Dioscorides makes the recommendation; Orange, Only Galen makes the recommendation. Numbers in cells correspond to the number of studies citing a plant taxon-use-category pair*.

GAS, gastrointestinal disorders (including liver and spleen); URO, urological problems; RES, respiratory complaints (including angina, sore throat, pleurisy); DER, dermatologic problems (including oral cavity, varicose veins, and hemorrhoids); SKM, skeleto-muscular disorders (including hematoma and gout); NER, central and peripheral nervous system (including headache, toothache, analgesic uses, epilepsy, insomnia); GYN, application in women's medicine (gynecology); FEV, fever, malaria; EYE, problems of the eye; EAR, problems of the ear; NOS, problems of the nose not related to respiratory diseases (epistaxis, polyps).

* *Eighth Book “Delle compositioni de medicamenti secondo i luoghi” by Galen (Matthioli, 1967–1970, pp. 535–536)*.

Of the 3′104 citations of all three South Italian regions and 87 taxa together, 78% correspond with Dioscorides and 55% with Galen, while 23% of the uses mentioned in Dioscorides and 14% of those in Galen are not represented in contemporary traditions. Gastrointestinal (81.9–92.3%), dermatological (75.8–96.5%), and urological applications (50–80.5%) of contemporary ethnomedical knowledge in the three regions coincide the most with the recommendations in Dioscorides and Galen. Recent plant uses related to women's medicine shows high concordance with Dioscorides (83.3–87.5%) but a poor overlap with Galen (17.5–37.5%). The importance of the use-categories in terms of the number of included plant taxa shows considerable differences between the classic Greco-Roman and modern sources. For 59 of the 87 taxa, Dioscorides makes reference to a use related to women's medicine in relation to only 24 in Campania, 25 in Sardinia and 29 in Sicily. Also the remedies for eye (34) and ear problems (28) is considerably higher in Dioscorides with respect to the three South Italian regions, with 9–13 and 2–9 taxa indicated, respectively. Using herbal remedies for fever is, on the contrary, a cultural trait more common with the contemporary data where 14 taxa are used in Campania, 21 in Sardinia and 16 in Sicily, while Dioscorides recommends 9 and Galen only 2 out of the 87 taxa. For an overview see Table 1 and Supplementary Tables S1–S3.

### Probit regression

The significance of the interaction between geography and joint recommendations is by far lower than that of the effect of geography taken alone. This means that the differences regarding citations proportions (or the number of use-citations relative to the number of examined papers) between regions are significant also for jointly recommended plant uses, but are less pronounced than the differences between regions considering all plant use combinations. Details are given in the Supplementary Material (Supplementary Table S5).

### Causal inference

**Figure 5:**
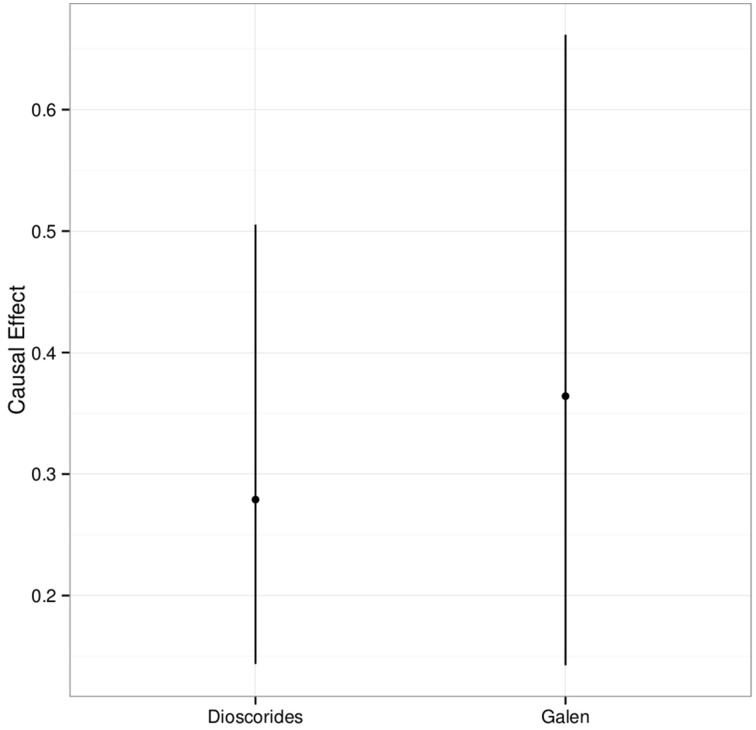
**Overall posterior distribution of the causal effect of Dioscorides and Galen on the contemporary plant use traits over all 87 taxa, 11 uses-categories and for all three regions together**. The plot shows the mean effect (point) along with the 95% credible interval.

The estimation of the causal effect is measured as the increment in the probability that a plant taxon is used in contemporary local herbal medicine for a specific use due to Dioscorides or Galen accounting for the confounding effects. The posterior distribution of the causal effect of the two authors for the 87 plant taxa over all three South Italian regions together is summarized in Figure 5. The mean effect α (point) along with its 95% credible intervals are reported. These intervals, although located at larger values, are compatible with the results obtained in our previous study (Leonti et al., 2010), and a causal effect of the two authors almost certainly exists for all three regions.

**Figure 6:**
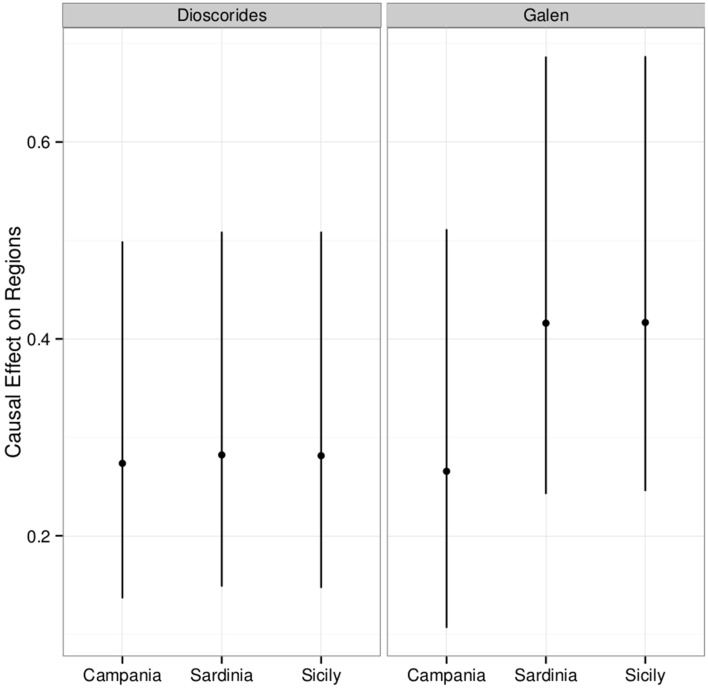
**Posterior distribution of the causal effect of Dioscorides and Galen on the contemporary plant use traits of the 87 taxa conditioned by the region of Campania, Sardinia, and Sicily**. The plot shows the mean effect (point) along with the 95% credible interval.

The overall causal effect of Dioscorides' *DMM* (*ex* Matthioli 1568) on the uses of the 87 plant taxa in the three regions is 27.6% (15.1–51.7%), while the effect of Galen's *DSMF* is 36.4% (14.3–66.2%). *DMM* causally influenced the medicinal use of the 87 plant taxa between 27.3 and 27.9% in all three regions (Figure 6). Galen causally influenced popular use of the 87 plant taxa of 26.6% in Campania, 41.6% in Sardinia and 41.7% in Sicily (Figure 6).

### Contemporary traits not coinciding with dioscorides and galen cross-checked with the content of a popular book on herbal medicine

Practically all of the most frequently reported contemporary plant use traits not mentioned in Dioscorides' and Galen's works are described in Reader's Digest (1980). Exceptions are few, such as applying parsley (*Petroselinum crispum*) for ear problems and the perceived analgesic properties of *Sambucus nigra*. In fact the emollient and diuretic properties of *Parietaria officinalis* are reported in the popular book, as well as its use against lithiasis and toothache (p. 223). *Rosmarinus officinalis* is recommended for sprains, asthma and cellulitis (p. 252) as well as hair loss (p. 381). The trait of using *Ruscus aculeatus* to treat hemorrhoids and varicose veins is reported on page 243, while the sedative properties of *Crataegus* sp. and its use to treat anxiety is described on page 78. The sedative and calming properties of *Malva sylvestris* and its use to treat bronchitis, asthma, cough, toothache, nervousness and as an eye cleanser, are referred to on page 192, while on page 261 *Sambucus nigra* is recommended for bronchitis and *Papaver rhoeas* praised for its beneficial effects against angina and bronchitis (p. 253). The application of *Tanacetum vulgare* against contusions and strains is recommended on page 283, and the usefulness of *Tamus communis* (syn.: *Dioscorea communis*) for arthritis and contusions is highlighted on page 282. The emmenagogue properties of *Senecio vulgaris* and its use in menstruation problems is explained on page 273, while the febrifuge properties of *Centaurium erythraea* and *Marrubium vulgare* are mentioned on page 106 and 194, respectively.

## Discussion

### General analysis

Overall, the present analysis is more reliable than our previous approach (Leonti et al., 2010) because we considered a larger amount of data and included an additional confounding variable. The intervals (Figures 5, 6), although located at larger values are compatible with our previous results (Leonti et al., 2010). A causal effect of the two authors exists almost for sure for all three regions. In our previous approach we estimated that one out of five plant uses stems directly from Matthioli's work (including the uses mentioned by Dioscorides as well as those recommended by Matthioli himself). This corresponded to a 20% average increment of the probability of finding a plant taxon mentioned for a certain use-category indicated by Matthioli during the sixteenth century. The results of the present analysis suggest, however, that around one in three (Galen) and one in four (Dioscorides) plant uses recorded in the three South Italian regions stem directly from the recommendations made by the two physicians some 2000 years ago. This corresponds to a slightly stronger effect than the one observed for Campania for a smaller set of taxa and the recommendations taken from *DMM* as well as Matthioli's comments.

We assume that the joint recommendations by Galen and Dioscorides equalize interregional citation proportions because of the causal effect we have found with the causal inference approach. In concordance with other studies (Hallpike, 1988; Guglielmino et al., 1995), our results suggest that the transmission of knowledge has been influenced more by cultural determinants than by ecological or geographical factors.

However, Dioscorides has not invented the tradition of writing herbals but was influenced by works of other scholars whom he cites in his work. Dioscorides has taken inspiration and instruction from Sextius Niger and Krateuas, and also from Iollas of Bithynia and Herakleides of Tarentum, while he quotes from works ascribed to Theophrastus and Hippocrates (Singer, 1927; Matthioli, 1967–1970). Galen, in his turn, has acknowledged Dioscorides' authority citing his name several times in *DSMF* (Galenus, 1561; Riddle, 1985). Overall, by reading Dioscorides' and Galen's texts it becomes clear that both report on a perceived cultural consensus of medicinal plant use enriched by their personal experiences.

Cavalli-Sforza et al. (1982) pointed out how oblique knowledge transmission through one or a few teachers creates an increase in trait homogeneity and allows for fast cultural change within a population, but at the same time may lead to greater variation between populations. An effect leading to a similar outcome has been described with models of cultural evolution, which suggest that natural selection favors psychological mechanisms that lead to conformist transmission influencing social learning behavior (Henrich and Boyd, 1998). Adaptive conformist transmission entails the adjustment and alignment of individuals' behavior in concordance with that of other group members, and in a cross-cultural context might explain the maintenance of between-group differences (Henrich and Boyd, 1998). Concerning the context of our research question, however, the three South Italian regions, (Campania, Sardinia, and Sicily), although having experienced distinct historic developments, have gradually grown into a more or less coherent cultural area since the Roman conquest. It can therefore be assumed that the scaling down of cultural barriers facilitated conformist transmission in Southern Italy and promoted the adoption of “new cultural traits.”

#### Impact of the scientific sphere on the frequency of popular plant use traits

External storage of human knowledge, such as writing, influences technological change, preserves knowledge and allows the transmission of knowledge between populations distant in time and space. At the same time populations adapt their pharmacopeia to the latest scientific progress trying to keep pace with the epidemiological situation and therapeutical needs. The advancements in pharmacology and epidemiology clearly allowed for a more Darwinian perspective on herbal medicine, including the isolation of pure biologically active principles. Globalization and modernization have led to new medical thinking, in both professional and popular spheres, moving away from Galenic humoral theory and the doctrine of signatures. In the course of the introduction of vaccinations and prescription drugs during the early twentieth century, herbal medicine lost its appeal and importance in the more industrialized countries. The turnaround that started some decades ago, accompanied by a changing epidemiology where cancers, cardiovascular and other chronic illnesses superseded infectious diseases, is culminating in the ever-growing popularity of nutraceuticals (Etkin, 2006).

The comparison of antique with contemporary plant uses suggests that hormonal birth control and systematic clinical controls considerably reduced the need and popularity of using herbal remedies in women's medicine. Likewise, herbal remedies are today rarely indicated for the treatment of sensory organs such as the eyes and ears in Southern Italy. For eyes and ears prescription drugs are generally preferred, such as isotonic and sterile eye drops as well as antibacterials, which -notwithstanding their precise indications, are used against all kind of infections, whether bacterial or not. This indiscriminate prescription and use of antibacterial drugs, however, afflicts Italy with a particularly high incidence of methicillin-resistant *Staphylococcus aureus* strains (Porretta et al., 2003; Tiemersma et al., 2004). In fact, the effectiveness of infection therapies are difficult to evaluate on a popular level, as proper immune response usually leads to the elimination of germs and the restoration of health. In such cases, the recovery of the patient may be ascribed to a remedy applied, which at best was ineffective. In this context it has been argued that therapeutically ineffective and therefore repeatedly practiced treatments have a greater chance of being copied and transmitted (Tanaka et al., 2009). However, rarer and less frequently mentioned plant uses such as those to treat eye and ear problems, might also have a higher chance of being replaced or abandoned with respect to more common applications due to random effects (Leonti, 2011). Thus, besides the introduction of real innovations and the creation of new cultural traits cultural changes can occur also through cognitively biased or incorrect knowledge transmission and random processes.

#### Exchange of cultural traits between the popular and the scientific sphere and the loss of local knowledge

Oral and written knowledge influence each other creating what has been described as a feedback loop of knowledge transmission mediated through cultural exchange between popular and science-based knowledge systems (Leonti, 2011). This process was -and is- far from straightforward, and involves modifications of traits, i.e., recombination of plant-uses, as well as the diffusion of completely new traits within the popular and scientific spheres. In terms of quality there is no need to divide between oral and written knowledge transmission (Totelin, 2009) although writing permits knowledge transmission with higher fidelity. However, written transmission of knowledge has a higher quantitative potential and can be traced back in time. It can be anticipated that with increasing magnitude the transmission of plant use traits lies within the domain of the written scientific sphere. Today, apart from biomedicine and evidence-based phytotherapy, a wealth of alternative treatment options, including different herbal medicinal systems, such as Ayurveda or Chinese Medicine, are widely available to the European citizen. This development is paralleled by the correct perception by ethnobotanists that plant use traits involving the local flora are becoming less important on the local level. We assume that high-fidelity knowledge transmission through scripts and advertisement has led to an overall diversification of plant use traits since antiquity at the expense of the medicinal importance of native floras. Ethnobotanists mourn this loss of importance of the local flora for medicinal purposes, claiming a pressing need for the documentation of the remaining knowledge arguing that such information is crucial for local health care, the development of herbal remedies and bioprospecting. However, considering that even contemporary plant uses in Southern Italy not discussed by Dioscorides and Galen can be found in a randomly chosen popular book on herbal medicine from the second half of the twentieth century, such argumentations appear to have a weak basis as most of the knowledge has already been documented. Where and when exactly these traits evolved and to which extent popular books on herbal medicine influenced local herbal practices, goes beyond the scope of this analysis. However, the traits not reported by Dioscorides and Galen are evidently well documented elsewhere.

### Cultural evolution and evidence-based medicine

The experimental search for effective natural remedies by individuals and populations is generally referred to as the “trial and error” approach. Especially the emergence of new epidemics (e.g., diabetes, cardiovascular disorders, HIV) and the introduction of “exotic” plant species, guides the popular experimentation with old and new remedies for new and old diseases. As herbal medicine and phytotherapy research are striving to build an evidence base, ethnopharmacology takes popular plant use traits or memes and subjects them to laboratory and clinical testing in order to select for the most beneficial traits. This aspect of cultural mutation and evolution is different from genetic evolution as it arises from problem solving and is directional, purposeful, and non-random with respect to its adaptive consequences (Kronfeldner, 2007; Cardoso and Atwell, 2011). The cognitive capacity of the human mind to select effective and appropriate applications of plants as medicine, adds to the cultural success of such traits or memes, as well as to the fitness of the human population adopting that trait. On the other hand, traits of plant use may become obsolete because scientific progress has led to the development of more effective treatments and preventions, or because the disease has been eradicated. In this sense, a quotation from R. Dawkins: “Nothing is more lethal to certain kinds of memes than a tendency to look for evidence” (1976, p. 198), describes perfectly the goal of evidence-based (herbal) medicine.

## Conclusion

We have provided further evidence for how repeated cumulative transmission of cultural traits through written sources shapes consensus on the use of medicinal plants. The teacher-like oblique transmission of Dioscorides' and Galen's medico-botanical treatises, exert a causal and homogenizing effect on contemporary medicinal plant use in Southern Italy. Cumulative knowledge derived from the repeated empirical testing of natural remedies over the past 2000 years in Southern Italy, Europe and elsewhere, have led to a selection of applications, memes or cultural traits with perceived favorable healing outcomes. Evidence-based medicine exposes anecdotal treatment reports to pharmacological testing, chemical analysis and clinical trials, in an attempt to falsify or verify the hypothesis of efficacy. The subsequent divulgence and commercial exploitation of the scientific data adds to the fitness of human cultures and, in a self-reinforcing process, leads to the emergence of new cultural traits at the local level, in turn leading to a homogenizing effect between local, global and scientific medical realities. We conclude that cultural interactions lead to new challenges, which can be approached in a most creative way by mixing cultural traits, which eventually helps to solve problems and may lead to innovation and progress.

## Author contributions

ML together with SC and MC designed the study; LC and PS collected the data and SC together with MC carried out the statistical analysis. ML wrote the paper and all authors commented on and approved the manuscript.

## Funding

The research leading to these results has received funding from the People Programme (Marie Curie Actions) of the European Union's 7th Framework programme FP7/2007/2013 under REA grant agreement no. PITN-GA-2013-606895–MedPlant.

### Conflict of interest statement

The authors declare that the research was conducted in the absence of any commercial or financial relationships that could be construed as a potential conflict of interest.

## Footnotes

^1^Kew Medicinal Plant Names Services (http://www.kew.org/mpns). (accessed: 14.08.2015).
