# Supplementary material for: From cumulative cultural transmission to evidence-based medicine: evolution of medicinal plant knowledge in Southern Italy
Source: Front Pharmacol. 2015 Sep 30;6:207. doi: 10.3389/fphar.2015.00207 (PMC4588697; doi:10.3389/fphar.2015.00207)
Supplement: Supplementary file 1 [file Table1.PDF]

Supplementary material including tables S1-S5,

for the publication entitled:

“From cumulative cultural transmission to evidence-based medicine: Evolution of medicinal plant knowledge in Southern Italy”

Marco Leonti[1*], Peter Staub[1], Stefano Cabras[2,3], Maria Eugenia Castellanos[4], Laura Casu[5]

[1]Department of Biomedical Sciences, University of Cagliari, 09124 Cagliari, Italy;
[2]Department of Mathematics and Informatics, University of Cagliari, 09124 Cagliari, Italy;
[3]Department of Statistics, Carlos III University of Madrid, 28903 Getafe, Spain;
[4]Department of Statistics and O.R. Rey Juan Carlos University, 28938 Móstoles, Spain;
[5]Department of Life and Environmental Sciences, University of Cagliari, 09124 Cagliari, Italy

Frontiers in Ethnopharmacology, September 2015, doi: 10.3389/fphar.2015.00207

Supplementary table 1. Contemporary plant uses in Campania compared to recommendations in Dioscorides' *DMM* (*ex* Matthioli, 1568) and Galen's *DSMF.*

CAMPANIAN DATABASE	GAS	DER	NER	SKM	GYN	RES	FEV	UR0	EAR	EYE	NOS
Adiantum capillus-veneris L.	1	2	0	0	4	5	0	0	0	0	0
Allium cepa L.	3	7	0	0	1	4	0	4	0	0	0
Anagallis arvensis L. s.l.	0	1	0	0	0	2	0	0	0	0	0
Anemone sp.	0	2	2	1	0	0	0	0	0	0	0
Apium sp.	0	0	0	0	0	1	0	2	0	0	0
Artemisia sp.	2	0	1	1	0	0	0	0	0	0	0
Arum sp.	0	0	0	2	0	0	0	0	0	0	0
Arundo sp.	1	1	0	0	0	1	0	1	0	0	0
Asparagus sp.	2	0	0	0	0	0	0	6	0	0	0
Asphodelus sp.	0	1	0	1	0	0	0	0	0	0	0
Avena sp.	0	1	1	0	0	1	0	0	0	0	0
Brassica sp.	3	3	0	2	0	2	1	0	0	0	0
Calamintha nepeta (L.) Savi s.l.	4	2	2	1	0	4	0	0	0	0	0
Centaurium erythraea Rafn. s.l.	2	1	1	0	0	0	3	0	0	0	0
Ceratonia siliqua L.	2	1	0	0	0	3	0	0	0	0	0
Ceterach officinarum Willd. s.l.	1	0	0	0	1	4	0	1	0	0	0
Cichorium intybus L. s.l.	4	2	1	1	0	0	1	1	0	0	0
Convolvulus arvensis L.	1	1	0	1	0	0	0	0	0	0	0
Crataegus sp.	1	0	3	1	0	2	1	0	0	0	0

Cyclamen sp.	1	0	0	0	0	0	0	0	0	0	0
Cydonia oblonga Mill.	0	1	0	0	0	1	1	0	0	0	0
Cynara cardunculus L. & *scolymus* L. s.l	5	0	0	0	0	0	0	1	0	0	0
Cynodon dactylon (L.) Pers.	5	0	1	1	0	4	1	7	0	0	0
Daucus carota L. s.l.	1	2	0	0	0	3	0	2	0	0	0
Ecballium elaterium (L.) A. Rich.	2	0	0	0	0	0	0	0	0	0	0
Equisetum sp.	0	3	0	0	0	0	0	1	0	0	0
Ficus carica L.	1	7	0	0	0	7	0	1	0	0	0
Foeniculum vulgare Mill.	6	1	1	0	1	3	0	1	0	0	0
Fumaria sp.	3	3	0	0	0	0	0	0	0	0	0
Hedera helix L. s.l.	0	4	2	2	1	3	0	0	0	0	0
Helichrysum italicum (Roth) G. Don s.l.	0	0	0	0	0	2	0	0	0	0	0
Helleborus sp.	0	0	1	1	2	0	0	0	0	0	0
Hordeum vulgare L.	0	1	0	0	0	4	0	2	0	0	0
Hypericum perforatum L. & *perfoliatum* L.	1	8	1	1	0	1	0	0	0	0	0
Juglans regia L.	5	3	1	1	0	3	0	1	0	0	0
Lactuca sp.	5	6	6	1	2	1	0	1	0	1	0
Laurus nobilis L.	10	2	3	4	1	6	2	1	1	0	0
Lavatera sp. & *Althaea* sp.	3	3	0	0	0	3	0	0	0	0	0
Linum usitatissimum L.	3	6	1	1	0	5	0	0	0	1	0
Lonicera sp.	0	1	0	0	0	0	0	1	0	0	0
Malva sp.	7	8	3	1	3	8	1	1	0	0	0
Marrubium vulgare L.	1	0	0	0	1	1	1	0	0	0	0
Matricaria chamomilla L. & *Tanacetum* sp.	8	5	8	5	3	3	0	1	1	1	0
Mentha pulegium L.	1	0	0	1	0	2	0	0	0	0	0
Mentha sp.	9	4	4	2	1	4	0	0	0	0	0
Morus sp.	1	0	0	0	0	2	0	1	0	0	0
Muscari sp.	0	0	1	0	0	0	0	2	0	0	0
Myrtus communis L.	2	2	0	2	1	1	0	1	0	1	0
Nasturtium officinale R. Br.	1	0	0	0	0	0	0	1	0	0	0

Ocimum basilicum L.	5	1	1	0	0	2	0	1	0	0	0
Olea europaea L.	4	6	0	3	1	2	1	1	1	0	0
Origanum vulgare L. s.l. & *majorana* L.	4	2	3	1	1	6	0	0	0	0	0
Papaver rhoeas L.	0	2	10	0	0	4	0	0	0	0	0
Papaver somniferum L.	0	0	2	0	0	0	0	0	0	0	0
Parietaria sp.	6	7	0	4	1	3	0	5	0	0	0
Petroselinum crispum (Mill.) Fuss	3	2	1	1	5	0	0	1	0	0	0
Pinus sp.	0	2	0	0	0	0	0	1	0	0	0
Pistacia lensticus L.	0	1	1	1	0	1	0	0	0	0	0
Pistacia terebinthus L.	0	1	0	0	0	1	0	0	0	0	0
Plantago sp. (*P. psyllium* not included)	2	9	0	2	0	3	0	3	0	1	0
Polygonum aviculare L. s.l.	4	0	0	1	0	1	0	2	0	0	0
Prunus cerasus L. & *avium* L.	1	0	0	1	0	3	0	3	0	0	0
Prunus dulcis (Mill.) D.A. Webb	3	0	0	0	0	2	0	0	0	0	0
Prunus persica (L.) Batsch	1	0	0	0	0	0	0	0	0	0	0
Punica granatum L.	4	0	1	1	1	0	1	1	0	0	0
Ranunculus sp.	0	1	1	1	0	0	0	0	0	0	0
Ricinus communis L.	2	1	0	0	0	0	0	0	0	0	0
Rosa sp.	2	4	0	0	0	1	0	1	0	1	0
Rosmarinus officinalis L.	5	1	2	2	1	4	0	0	0	0	0
Rubus sp. (*R. idaeus* not included)	4	7	0	0	1	5	0	2	0	0	0
Rumex sp.	2	4	0	0	0	0	0	0	0	0	0
Ruscus sp.	1	1	0	0	0	0	0	6	0	0	0
Ruta sp.	5	4	4	7	3	0	0	0	1	1	0
Sambucus nigra L.	7	6	2	3	0	6	1	3	1	1	0
Senecio sp.	0	0	0	1	2	0	0	1	0	0	0
Solanum nigrum L.	0	2	1	1	0	0	0	0	0	0	0
Sonchus sp.	1	2	0	0	0	1	0	1	0	0	0
Tamus communis L. (now: *Dioscorea communis* (L.) Caddick & Wilkin.)	0	0	0	2	0	0	0	0	0	0	0

Thymus sp.	3	2	0	1	0	4	0	1	0	1	0
Trigonella foenum-graecum L.	0	0	0	0	1	1	0	0	0	0	0
Triticum sp.	1	2	2	0	1	1	0	0	0	0	0
Tussilago farfara L.	0	2	1	0	0	4	0	0	0	0	0
Ulmus sp.	0	4	0	0	0	1	1	0	0	0	0
Umbilicus sp.	0	3	0	0	0	0	0	0	0	0	0
Urtica sp.	2	8	1	8	0	2	0	3	0	0	0
Verbascum sp.	0	2	1	0	0	4	0	0	0	0	0
Verbena officinalis L.	2	1	1	1	0	1	2	0	0	0	0
**TOTAL**	**182**	**185**	**79**	**76**	**40**	**159**	**18**	**77**	**5**	**9**	**0**

Red = Dioscorides and Galen recommend the use; Yellow: Only Dioscorides makes the recommendation; Orange: Only Galen makes the recommendation. Numbers in cells correspond to the number of studies citing a plant taxon-use category-pair.

Supplementary table 2. Contemporary plant uses in Sardinia compared to recommendations in Dioscorides' *DMM* (*ex* Matthioli, 1568) and Galen's *DSMF.*

SARDINIAN DATABASE	GAS	DER	NER	SKM	GYN	RES	FEV	UR0	EAR	EYE	NOS
Adiantum capillus-veneris L.	1	5	4	0	5	5	0	3	0	1	0
Allium cepa L.	2	4	1	1	0	5	0	4	2	0	0
Anagallis arvensis L. s.l.	0	1	1	0	0	1	0	0	0	0	0
Anemone sp.	1	0	0	0	1	0	0	1	0	0	0
Apium sp.	4	1	0	2	0	1	1	2	0	0	0
Artemisia sp.	11	2	3	3	1	6	1	1	0	1	0
Arum sp.	0	6	0	2	0	1	0	1	0	0	0
Arundo sp.	0	7	0	0	2	0	0	2	1	0	0
Asparagus sp.	0	0	2	2	0	1	0	7	0	0	0
Asphodelus sp.	0	12	0	1	0	4	1	2	0	0	0
Avena sp.	2	1	0	0	0	0	0	2	0	0	0
Brassica sp.	1	4	1	1	0	1	0	0	0	0	0
Calamintha nepeta (L.) Savi s.l.	1	0	0	0	0	0	0	0	0	0	0
Centaurium erythraea Rafn. s.l.	4	4	0	0	1	0	7	0	0	0	0
Ceratonia siliqua L.	4	0	0	0	0	1	0	0	0	0	0
Ceterach officinarum Willd. s.l.	3	1	0	0	0	0	1	3	0	0	0
Cichorium intybus L. s.l.	9	0	0	2	0	0	2	3	0	0	0
Convolvulus arvensis L.	3	1	0	0	0	0	0	0	0	0	0

Crataegus sp.	4	2	9	1	2	0	2	3	0	0	0
Cyclamen sp.	1	1	0	0	1	0	0	0	0	0	0
Cydonia oblonga Mill.	3	1	1	0	0	2	0	0	0	0	0
Cynara cardunculus L. & *scolymus* L. s.l	4	0	0	0	0	0	0	0	0	0	0
Cynodon dactylon (L.) Pers.	6	1	0	1	0	3	1	8	0	0	0
Daucus carota L. s.l.	4	2	2	0	2	1	0	3	0	0	0
Ecballium elaterium (L.) A. Rich.	3	0	1	0	0	0	0	0	0	0	0
Equisetum sp.	2	6	0	3	0	2	0	7	0	0	1
Ficus carica L.	4	8	1	1	0	4	0	0	0	0	0
Foeniculum vulgare Mill.	10	0	0	1	3	2	0	3	0	1	0
Fumaria sp.	5	3	1	0	0	2	0	1	0	0	0
Hedera helix L. s.l.	1	6	3	3	0	5	0	0	0	0	0
Helichrysum italicum (Roth) G. Don s.l.	1	3	2	3	0	4	0	0	0	0	0
Helleborus sp.	0	1	1	0	0	0	0	0	0	0	0
Hordeum vulgare L.	4	1	0	0	0	3	0	0	0	1	0
Hypericum perforatum L. & *perfoliatum* L.	3	9	2	4	1	2	0	2	0	0	0
Juglans regia L.	1	2	1	0	0	0	0	0	0	0	0
Lactuca sp.	0	1	0	0	0	0	0	0	0	0	0
Laurus nobilis L.	16	2	1	6	1	7	0	1	0	0	0
Lavatera sp. & *Althaea* sp.	3	5	0	1	0	6	0	0	0	2	0
Linum usitatissimum L.	5	3	1	1	1	8	0	0	1	0	0
Lonicera sp.	3	1	0	3	1	1	0	1	0	0	0
Malva sp.	19	18	3	0	2	12	1	7	0	3	0
Marrubium vulgare L.	10	2	3	5	3	8	5	0	0	0	0
Matricaria chamomilla L. & *Tanacetum* sp.	8	4	5	3	2	2	1	0	0	5	0
Mentha pulegium L.	4	2	1	1	0	0	0	1	0	0	
Mentha sp.	11	5	3	2	1	2	1	0	0	0	
Morus sp.	1	0	0	0	0	0	0	0	0	0	0
Muscari sp.	0	0	0	0	0	0	0	1	0	0	0
Myrtus communis L.	9	7	1	0	0	7	0	2	0	0	0

Nasturtium officinale R. Br.	7	3	2	0	1	4	1	4	0	0	0
Ocimum basilicum L.	5	1	3	0	0	3	0	0	1	0	0
Olea europaea L.	8	9	0	2	0	0	5	1	4	0	0
Origanum vulgare L. s.l. & *majorana* L.	3	0	1	0	0	0	0	0	0	0	0
Papaver rhoeas L.	1	0	9	1	0	5	0	0	0	0	0
Papaver somniferum L.	1	0	1	0	0	1	0	0	0	0	0
Parietaria sp.	12	8	6	1	0	4	2	13	1	0	0
Petroselinum crispum (Mill.) Fuss	8	2	3	0	2	1	0	5	1	1	0
Pinus sp.	0	1	0	0	0	1	0	0	0	0	0
Pistacia lensticus L.	6	11	2	3	0	4	0	0	0	0	0
Pistacia terebinthus L.	1	2	2	0	0	3	0	1	0	0	0
Plantago sp. (*P. psyllium* not included)	6	6	0	2	1	2	0	4	0	1	0
Polygonum aviculare L. s.l.	1	1	0	0	0	0	1	0	0	0	0
Prunus cerasus L. & *avium* L.	3	0	0	1	0	2	0	3	0	0	0
Prunus dulcis (Mill.) D.A. Webb	3	2	0	0	0	2	0	0	0	0	0
Prunus persica (L.) Batsch	1	0	0	0	0	0	0	0	0	0	0
Punica granatum L.	3	0	0	0	0	0	0	0	0	0	0
Ranunculus sp.	0	2	0	2	0	1	0	0	0	0	0
Ricinus communis L.	2	0	0	0	0	0	0	0	0	0	0
Rosa sp.	5	0	0	0	1	2	0	1	0	2	0
Rosmarinus officinalis L.	11	5	4	3	0	7	0	3	0	0	0
Rubus sp. (*R. idaeus* not included)	10	8	1	0	1	1	0	2	0	1	0
Rumex sp.	3	6	0	1	0	1	2	5	0	0	0
Ruscus sp.	2	4	2	4	0	0	0	5	0	0	0
Ruta sp.	8	1	1	2	1	1	1	0	1	5	0
Sambucus nigra L.	4	6	4	4	0	4	0	0	0	8	0
Senecio sp.	1	0	0	0	3	0	0	1	0	0	0
Solanum nigrum L.	0	5	5	3	0	2	0	0	0	0	0
Sonchus sp.	2	4	0	1	0	0	0	2	0	0	0
Tamus communis L. (now: *Dioscorea*	0	1	1	5	0	0	0	0	0	0	0

communis (L.) Caddick & Wilkin.)											
Thymus sp.	6	3	4	1	0	7	1	1	0	0	0
Trigonella foenum-graecum L.	0	0	0	0	0	1	0	0	0	0	0
Triticum sp.	2	5	2	3	0	2	0	0	0	0	0
Tussilago farfara L.	0	1	0	1	0	1	0	0	0	0	0
Ulmus sp.	0	1	0	1	0	0	0	0	0	0	0
Umbilicus sp.	2	10	1	0	0	1	0	2	0	0	0
Urtica sp.	10	16	4	6	2	3	1	8	1	0	2
Verbascum sp.	3	3	0	0	0	3	0	0	0	0	0
Verbena officinalis L.	1	1	2	0	0	0	2	0	0	0	0
**TOTAL**	**332**	**273**	**114**	**100**	**42**	**178**	**40**	**132**	**13**	**32**	**3**

Red = Dioscorides and Galen recommend the use; Yellow: Only Dioscorides makes the recommendation; Orange: Only Galen makes the recommendation. Numbers in cells correspond to the number of studies citing a plant taxon-use category-pair.

Supplementary table 3. Contemporary plant uses in Sicily compared to recommendations in Dioscorides' *DMM* (*ex* Matthioli, 1568) and Galen's *DSMF*.

SICILIAN DATABASE	GAS	DER	NER	SKM	GYN	RES	FEV	URO	EAR	EYE	NOS
Adiantum capillus-veneris L.	2	3	0	1	9	3	0	0	0	0	0
Allium cepa L.	2	2	1	1	0	3	0	4	0	0	1
Anagallis arvensis L. s.l.	0	1	0	0	0	2	0	0	0	1	0
Anemone sp.	0	0	1	0	0	0	0	0	0	0	0
Apium sp.	3	1	0	2	0	3	0	7	0	0	0
Artemisia sp.	10	8	0	0	1	2	4	1	0	2	0
Arum sp.	0	2	0	1	0	0	0	0	0	0	0
Arundo sp.	0	8	1	1	0	0	1	2	0	0	0
Asparagus sp.	3	0	0	1	0	1	0	6	0	0	0
Asphodelus sp.	0	12	0	1	0	0	0	0	0	0	0
Avena sp.	2	2	0	1	0	1	0	2	0	0	0
Brassica sp.	3	1	0	2	2	4	0	1	0	0	0
Calamintha nepeta (L.) Savi s.l.	5	6	3	3	1	1	0	1	0	0	0
Centaurium erythraea Rafn. s.l.	1	0	0	0	0	0	2	0	0	0	0
Ceratonia siliqua L.	3	0	0	0	0	8	0	1	0	0	0
Ceterach officinarum Willd. s.l.	0	1	1	0	0	1	0	11	0	0	0
Cichorium intybus L. s.l.	13	0	0	0	0	0	0	5	0	0	0

Convolvulus arvensis L.	3	2	0	2	0	0	0	0	0	0	0
Crataegus sp.	2	1	1	0	0	0	2	1	0	0	0
Cyclamen sp.	1	3	0	1	1	0	0	0	0	0	0
Cydonia oblonga Mill.	4	3	1	0	0	0	0	0	0	0	0
Cynara cardunculus L. & *scolymus* L. s.l	5	1	0	0	0	1	0	1	0	0	0
Cynodon dactylon (L.) Pers.	7	2	0	0	1	1	0	17	0	0	0
Daucus carota L. s.l.	3	2	0	0	1	1	0	2	0	1	0
Ecballium elaterium (L.) A. Rich.	3	3	2	2	1	0	2	0	0	0	0
Equisetum sp.	2	2	0	0	0	1	0	9	0	0	2
Ficus carica L.	2	7	0	0	1	7	0	0	0	0	0
Foeniculum vulgare Mill.	11	2	0	0	3	3	0	4	0	1	0
Fumaria sp.	1	1	1	0	1	0	0	2	0	0	0
Hedera helix L. s.l.	1	8	1	2	1	2	0	0	0	0	0
Helichrysum italicum (Roth) G. Don s.l.	1	1	0	0	0	1	0	0	0	0	0
Helleborus sp.	0	1	1	0	0	0	0	0	0	0	0
Hordeum vulgare L.	3	1	0	0	0	2	0	1	0	0	0
Hypericum perforatum L. & *perfoliatum* L.	1	11	1	3	0	2	0	1	0	0	0
Juglans regia L.	1	2	0	0	0	0	0	0	0	0	0
Lactuca sp.	2	4	4	1	0	0	0	1	0	0	0
Laurus nobilis L.	18	2	2	3	2	4	1	1	0	0	0
Lavatera sp. & *Althaea* sp.	2	1	0	0	0	0	0	1	0	0	0
Linum usitatissimum L.	2	4	1	0	0	2	0	0	0	0	0
Lonicera sp.	0	0	1	0	0	1	0	0	0	0	0
Malva sp.	14	10	2	0	0	4	0	6	0	2	0
Marrubium vulgare L.	1	1	1	2	0	4	1	0	0	0	0
Matricaria chamomilla L. & *Tanacetum* sp.	3	1	3	1	2	0	0	0	0	2	0
Mentha pulegium L.	4	2	2	0	1	3	0	0	0	1	0
Mentha sp.	5	2	3	1	1	3	1	0	0	0	0
Morus sp.	2	1	0	0	0	2	1	1	0	0	0

Muscari sp.	1	2	0	0	0	0	0	2	0	0	0
Myrtus communis L.	1	5	2	0	1	1	0	1	0	0	0
Nasturtium officinale R. Br.	3	0	0	0	2	2	0	2	0	0	0
Ocimum basilicum L.	1	2	1	0	1	1	0	0	0	0	0
Olea europaea L.	1	2	1	1	0	1	1	0	0	0	0
Origanum vulgare L. s.l. & *majorana* L.	3	1	3	4	1	7	0	1	0	0	0
Papaver rhoeas L.	1	0	11	0	0	3	1	0	0	0	0
Papaver somniferum L.	0	0	2	0	0	0	0	0	0	0	0
Parietaria sp.	9	11	2	5	0	2	1	13	0	1	0
Petroselinum crispum (Mill.) Fuss	3	1	1	0	1	0	0	4	1	0	1
Pinus sp.	0	2	1	0	0	2	0	1	0	0	0
Pistacia lensticus L.	0	2	1	0	0	2	2	0	0	0	0
Pistacia terebinthus L.	1	1	0	2	0	0	0	0	0	0	0
Plantago sp. (*P. psyllium* not included)	2	5	0	1	0	0	0	1	0	1	0
Polygonum aviculare L. s.l.	1	4	0	0	0	0	0	7	0	0	0
Prunus cerasus L. & *avium* L.	2	0	0	1	0	0	0	1	0	0	0
Prunus dulcis (Mill.) D.A. Webb	2	1	0	0	0	1	0	0	0	0	0
Prunus persica (L.) Batsch	2	1	1	0	0	0	0	1	0	0	0
Punica granatum L.	4	1	0	0	1	2	0	1	0	0	0
Ranunculus sp.	0	3	0	2	0	0	0	0	0	0	0
Ricinus communis L.	3	1	1	1	1	0	0	0	0	0	0
Rosa sp.	1	1	0	0	0	1	0	1	0	2	0
Rosmarinus officinalis L.	6	0	3	3	1	2	0	0	0	0	0
Rubus sp. (*R. idaeus* not included)	5	13	0	0	0	0	0	1	0	0	0
Rumex sp.	1	3	2	0	0	0	0	0	0	0	0
Ruscus sp.	1	2	0	0	0	0	0	2	0	0	0
Ruta sp.	15	2	2	8	4	1	0	1	0	0	0
Sambucus nigra L.	2	4	2	7	0	3	1	2	1	0	0
Senecio sp.	3	1	0	0	1	1	0	0	0	0	0
Solanum nigrum L.	0	5	2	2	0	0	0	1	0	0	0

Sonchus sp.	5	1	2	1	0	0	0	1	0	0	0
Tamus communis L. (now: *Dioscorea communis* (L.) Caddick & Wilkin.)	0	0	0	3	0	0	0	1	0	0	0
Thymus sp.	3	2	0	1	1	5	0	0	0	0	0
Trigonella foenum-graecum L.	1	1	1	0	0	0	0	1	0	0	0
Triticum sp.	1	3	0	0	0	0	0	0	0	0	0
Tussilago farfara L.	0	5	0	0	0	4	1	0	0	0	0
Ulmus sp.	0	3	0	3	0	0	0	0	0	0	0
Umbilicus sp.	0	10	1	0	0	0	0	0	0	0	0
Urtica sp.	7	12	0	3	1	1	0	1	0	0	1
Verbascum sp.	0	11	1	0	0	1	0	1	0	0	0
Verbena officinalis L.	3	4	3	6	1	2	4	0	0	0	0
**TOTAL**	**245**	**256**	**80**	**86**	**46**	**118**	**26**	**137**	**2**	**14**	**5**

Red = Dioscorides and Galen recommend the use; Yellow: Only Dioscorides makes the recommendation; Orange: Only Galen makes the recommendation. Numbers in cells correspond to the number of studies citing a plant taxon-use category-pair.

Supplementary table 4. List of plant species treated as ethnotaxa and considered in the analysis.

Nr.	Taxon	Family
**1**	*Adiantum capillus-veneris* L.	Adiantaceae
**2**	*Allium cepa* L.	Amaryllidaceae
**3**	*Anagallis arvensis* L. s.l.	Primulaceae
**4**	*Anemone* spp. (*A. coronaria* L., *A. hortensis* L., *A. nemorosa* L.)	Ranunculaceae
**5**	*Apium graveolens* L. & *A. nodiflorum* (L.) Lag.	Apiaceae
**6**	*Artemisia* spp. (*A. alba* Turra, *A. abrotanum* L., *A. arborescens* (Vaill.) L.)	Asteraceae
**7**	*Arum* spp. (*A. italicum* Mill., *A. maculatum* L., *A. pictum* L.f.)	Araceae
**8**	*Arundo donax* L. & *A. plinii* Turra	Poaceae
**9**	*Asparagus* spp. (*A. acutifolius* L., *A. albus* L., *A. officinalis* L.)	Asparagaceae
**10**	*Asphodelus* spp. (*A. albus* Mill., *A. cerasiferus* J. Gay, *A. fistulosus* L., *A. macrocarpus* Parl., *A. microcarpus* Salzm. & Viv., *A. ramosus* L.)	Xanthorrhoeaceae
**11**	*Avena* spp. (*A. barbata* Pott ex Link, *A. fatua* L., *A. sativa* L.)	Poaceae
**12**	*Brassica* spp. (*B. oleracea* L., *B. napus* L., *B. rapa* L.)	Brassicaceae
**13**	*Calamintha nepeta* (L.) Savi s.l.	Lamiaceae
**14**	*Centaurium erythraea* Rafn. s.l.	Gentianaceae
**15**	*Ceratonia siliqua* L.	Fabaceae
**16**	*Ceterach officinarum* Willd. s.l.	Aspleniaceae
**17**	*Cichorium intybus* L. s.l.	Asteraceae
**18**	*Convolvulus arvensis* L.	Convolvulaceae
**19**	*Crataegus monogyna* Jacq. & *C. laevigata* (Poir.) DC.	Rosaceae
**20**	*Cyclamen hederifolium* Aiton & *C. repandum* Sm	Primulaceae
**21**	*Cydonia oblonga* Mill.	Rosaceae
**22**	*Cynara cardunculus* L. & *C. scolymus* L. s.l	Asteraceae
**23**	*Cynodon dactylon* (L.) Pers.	Poaceae

**24**	*Daucus carota* L. s.l.	Apiaceae
**25**	*Ecballium elaterium* (L.) A. Rich.	Cucurbitaceae
**26**	*Equisetum* spp. (*E. arvense* L., *E. giganteum* L., *E. telmateia* Ehrh., *E. ramosissimum* Desf.)	Equisetaceae
**27**	*Ficus carica* L.	Moraceae
**28**	*Foeniculum vulgare* Mill.	Apiaceae
**29**	*Fumaria* spp. (*F. agraria* Lag., *F. capreolata* L., *F. officinalis* L., *F. parviflora* Lam.)	Papaveraceae
**30**	*Hedera helix* L. s.l.	Araliaceae
**31**	*Helichrysum italicum* (Roth) G. Don s.l.	Asteraceae
**32**	*Helleborus* spp. (*H. bocconei* Ten., *H. foetidus* L., *H. lividus* Aiton ex Curtis)	Ranunculaceae
**33**	*Hordeum vulgare* L.	Poaceae
**34**	*Hypericum perforatum* L. & *H. perfoliatum* L.	Hypericaceae
**35**	*Juglans regia* L.	Juglandaceae
**36**	*Lactuca* spp. (*L. sativa* L., *L. serriola* L., *L. viminea* (L.) J.Presl & C.Presl, *L. virosa* Habl.)	Asteraceae
**37**	*Laurus nobilis* L.	Lauraceae
**38**	*Lavatera* spp. (*L. cretica* L., *L. olbia* L., *L. trimestris* L.) & *Althaea officinalis* L.	Malvaceae
**39**	*Linum usitatissimum* L.	Linaceae
**40**	*Lonicera implexa* Aiton	Caprifoliaceae
**41**	*Malva* spp. (*M. neglecta* Wallr., *M. nicaeensis* All., *M. parviflora* L., *M. sylvestris* L.)	Malvaceae
**42**	*Marrubium vulgare* L.	Lamiaceae
**43**	*Matricaria chamomilla* L., *Tanacetum parthenium* (L.) Sch.Bip. & *T. vulgare* L.	Asteraceae
**44**	*Mentha pulegium* L.	Lamiaceae
**45**	*Mentha* spp. (*M. aquatica* L., *M. × piperita* L., *M. spicata* L., *M. suaveolens* Ehrh.)	Lamiaceae
**46**	*Morus alba* L. & *M. nigra* L.	Moraceae
**47**	*Muscari racemosum* Mill. & *Leopoldia comosa* (L.) Parl.	Asparagaceae
**48**	*Myrtus communis* L.	Myrtaceae
**49**	*Nasturtium officinale* R. Br.	Brassicaceae
**50**	*Ocimum basilicum* L.	Lamiaceae
**51**	*Olea europaea* L.	Oleaceae

**52**	*Origanum vulgare* L. s.l. & *O. majorana* L.	Lamiaceae
**53**	*Papaver rhoeas* L.	Papaveraceae
**54**	*Papaver somniferum* L.	Papaveraceae
**55**	*Parietaria* spp. (*P. judaica* L., *P. lusitanica* L., *P. officinalis* L.*)*	Urticaceae
**56**	*Petroselinum crispum* (Mill.) Fuss	Apiaceae
**57**	*Pinus halepensis* Mill. & *P. pinea* L.	Pinaceae
**58**	*Pistacia lensticus* L.	Anacardiaceae
**59**	*Pistacia terebinthus* L.	Anacardiaceae
**60**	*Plantago* spp. (*P. coronopus* L., *P. lagopus* L., *P. lanceolata* L., *P. major* L., *P. serraria* L.)	Plantaginaceae
**61**	*Polygonum aviculare* L. s.l.	Polygonaceae
**62**	*Prunus cerasus* L. & *P. avium* L.	Rosaceae
**63**	*Prunus dulcis* (Mill.) D.A. Webb	Rosaceae
**64**	*Prunus persica* (L.) Batsch	Rosaceae
**65**	*Punica granatum* L.	Lythraceae
**66**	*Ranunculus* spp. (*R. arvensis* L., *R. bulbosus* L., *R. paludosus* Poir., *R. muricatus* L., *R. millefolius* Banks & Sol., *R. sardous* Crantz, *R. sceleratus* L.) & *Ficaria verna* Huds.	Ranunculaceae
**67**	*Ricinus communis* L.	Euphorbiaceae
**68**	*Rosa* spp. (*R. canina* L., *R. sempervirens* L., *R. serafini* Viv.)	Rosaceae
**69**	*Rosmarinus officinalis* L.	Lamiaceae
**70**	*Rubus* spp.	Rosaceae
**71**	*Rumex* spp. (*R. acetosa* L., *R. acetosella* L., *R. bucephalophorus* L., *R. conglomeratus* Murray, *R. crispus* L., *R. obtusifolius* L., *R. pulcher* L., *R. sanguineus* L., *thyrsoides* Desf.)	Polygonaceae
**72**	*Ruscus aculeatus* L. & *R. hypoglossum* L.	Asparagaceae
**73**	*Ruta chalaepensis* L. & *R. graveolens* L.	Rutaceae
**74**	*Sambucus nigra* L.	Adoxaceae
**75**	*Senecio* spp. (*S. delphinifolius* Rchb., *S. lycopifolius* Desf., *S. vulgaris* L.) & *Jacobaea candida* (C.Presl) B.Nord. & Greuter	Asteraceae
**76**	*Solanum nigrum* L.	Solanaceae

**77**	*Sonchus* spp. (*S. asper* (L.) Hill, *S. oleraceus* (L.) L., *S. tenerrimus* L.)	Solanaceae
**78**	*Tamus communis* L. (now: *Dioscorea communis* (L.) Caddick & Wilkin.)	Dioscoreaceae
**79**	*Thymus herba-barona* Loisel., *T. vulgaris* L. & *Thymbra capitata* (L.) Cav.	Lamiaceae
**80**	*Trigonella foenum-graecum* L.	Fabaceae
**81**	*Triticum aestivum* L. & *T. durum* Desf.	Poaceae
**82**	*Tussilago farfara* L.	Asteraceae
**83**	*Ulmus glabra* Huds. & *U. minor* Mill.	Ulmaceae
**84**	*Umbilicus horizontalis* (Guss.) DC. & *U. rupestris* (Salisb.) Dandy	Crassulaceae
**85**	*Urtica* spp. (*U. atrovirens* Req. ex Loisel., *U. dioica* L., *U. membranacea* Poir. ex Savigny, *U. pilulifera* L., *U. urens* L.)	Urticaceae
**86**	*Verbascum* spp. (*V. creticum* (L.f.) Cav., *V. densiflorum* Bertol., *V. pulverulentum* Vill., *V. sinuatum* L., *V. thapsus* L.)	Scrophulariaceae
**87**	*Verbena officinalis* L.	Verbenaceae

Supplementary table 5. ANOVA table of Probit regression

	Df	Deviance	Resid.	Df Resid. Dev	Pr(>Chi)
**NULL**			2870	41.772	
**plant**	86	5.3002	2784	36.472	< 2e-16 ***
**Use**	10	11.0159	2774	25.456	< 2e-16 ***
**joint.rec**	1	1.3826	2773	24.073	< 2e-16 ***
**Geo**	2	2.4393	2771	21.634	< 2e-16 ***
**joint.rec:geo**	2	0.0790	2769	21.555	0.00626 **

Signif. codes: 0 ‘***’ 0.001 ‘**’ 0.01 ‘*’ 0.05 ‘.’ 0.1 ‘ ’ 1
